# Early-life antibiotics and childhood allergy: a multi-center cohort

**DOI:** 10.1186/s13223-026-01013-5

**Published:** 2026-02-03

**Authors:** Moath Hattab, Yaman Abu Sarrees, Mahmoud Sous, Mosab Najajrah, Hamza Karmi, Maysa Alawneh, Suhaib Hattab

**Affiliations:** 1 Department of Medicine, Faculty of Medicine and Allied Medical Sciences, An-Najah National University, Nablus, Palestine; 2 Faculty of Medicine, Al-Quds University, Jerusalem, Palestine; 3 Physiology, Pharmacology and Toxicology Division, Department of Biomedical Sciences and Preclinical Skills, Faculty of Medicine and Allied Medical Sciences, An-Najah National University, Nablus, Palestine

**Keywords:** Anti-bacterial agent, Infant, Allergy and immunology, Microbiota, Cohort studies

## Abstract

**Background:**

Antibiotic use in infants is hypothesized to alter the gut microbiota, influencing immune system dysregulation and increasing allergy risk. We aim to assess the prevalence of allergic diseases in children treated with different classes of antibiotics in early life.

**Methods:**

A retrospective cohort study was conducted from April 2024 to January 2025 in three main hospitals in the West Bank in Palestine. Records of pediatric admissions of children who received antibiotic treatment within their first six months of life were reviewed, followed by parents’ interview regarding the development of allergies.

**Results:**

A total of 423 medical records were included. The average age of children was 7.33 ± 1.38 years (mean ± SD), and 62.41% of them were males. The total prevalence of allergic diseases was 29.55%. Common manifestations of allergies were skin reactions (70.4%), wheezing (16.8%), and respiratory symptoms (10.4%). Among the most common reported triggers were food (10.17%) and dust (7.33%). The most commonly prescribed antibiotics were Beta-lactams; cefotaxime (78.49%), and ampicillin (63.59%). No statistically significant association was found between the number of antibiotics used and the development of allergies (*p* = 0.45). Similarly, different classes of antibiotics did not show an impact on developing allergies except for Trimethoprim/Sulfamethoxazole (*p* = 0.05). A significant decrease in allergy was observed with increasing age (*p* = 0.011).

**Conclusion:**

Allergic conditions affect about one third of children treated with antibiotics in early life. While allergic conditions tended to decrease with age, no association was observed between antibiotic number/class and later allergy, except for a hypothesis-generating signal toward lower odds with TMP-SMX.

## Highlights

- What is already known about this topic?
  Early-life antibiotic use is suspected to disrupt the gut microbiota, potentially increasing the risk of developing allergic diseases in childhood.
- What does this article add to our knowledge?
  In this first Palestinian cohort, ≈30% of children exposed to antibiotics in early life developed allergies; no class-specific link was found except a hypothesis-generating potential protective effect of TMP-SMX.
- How does this study impact current management guidelines?
  It supports antibiotic stewardship in infancy by discouraging unnecessary use, without implicating specific antibiotic classes as allergy triggers; highlights need for further research on TMP-SMX’s potential immunomodulatory effects.

## Key message

In this multi-hospital cohort of 423 children exposed to antibiotics in the first six months of life, ~ 30% developed allergic disease, with no association between number/class of antibiotics and later allergy; TMP-SMX showed a signal toward lower odds of allergy. Findings support antibiotic stewardship without implicating specific classes in increased allergy risk and highlight the need for prospective controlled trials.

## Introduction

Allergy is defined as a hypersensitive immune reaction to a specific environmental substance (the allergen). It can occur in all age groups with different causative agents and manifestations for each group [1]. Food allergies and atopic dermatitis are the most common allergic conditions to occur in infants & young children. Food allergies (from milk, egg, peanut, tree nuts, shellfish, and fish) present with a wide range of symptoms from urticaria to anaphylaxis, while atopic dermatitis distinct with eczematous lesions and pruritus that may change in the course of the disease [2, 3]. On the other hand, asthma is the dominant form of allergy in older children [4].

One of the most widely used drugs, particularly for pediatric patients, are antibiotics. Antimicrobials are often prescribed, regardless of whether they are used appropriately or not [5, 6]. Antibiotic exposure is common in the early years of life, even before birth such as intrapartum prophylaxis for Group-B Streptococcus for vaginal delivery, or antibiotic prophylaxis during cesarean section to avoid wound infection or during the first months of life as a treatment for bacterial infections (e.g.: urinary tract infections, bacterial meningitis and pneumonia) [7].

An appropriate development towards the physiological balance of the host is dependent on the microbial stimulus provided by colonization of the newborn digestive tract. A number of variables contribute to this colonization, which starts with facultative anaerobes and progresses to anaerobic genera. Thus, it is crucial to understand the variables that affect how the infant's microbiota establishes itself. The actively forming newborn gut microbiota raises with breastfeeding, but is also related to both short- and long-term disruptions by antibiotics. Because changes in the gut microbiota at this time may impair immunologic and metabolic development, it might have a significant effect on the patient’s health and illness throughout life [8–12].

Early-life gut microbiota influences immune maturation through effects on epithelial barrier integrity, microbial metabolite production (including short-chain fatty acids), and antigen presentation. Dysbiosis during infancy may reduce tolerogenic signaling and regulatory T-cell (Treg) expansion, while promoting a relative T-helper 2 (Th2) skew and IgE sensitization, thereby increasing susceptibility to atopic dermatitis, wheeze/asthma phenotypes, and food allergy. Antibiotics can disrupt colonization trajectories during this critical window and may therefore be associated with later allergic manifestations. [8–12]

Few studies have reported the relationship between infant antibiotic use and the development of allergic diseases such as asthma and food allergy, but our research, will be one of the first research on the topic in the Arab region, while also focusing on the class of antibiotic used during the first six months of life to find any association [13, 14]. We aimed to estimate the prevalence of allergic disease among children hospitalized and treated with different classes of antibiotics during the first 6 months of life in Palestine, and identify the factors associated with allergic disease among these children, and assess the safety and the risks of different classes of antibiotics associated with allergic disease, in order to provide evidence-based recommendations for health care professionals on the optimal choice of antibiotics in this age group.

## Methods

### Study design and setting

This was a retrospective cohort study, and was conducted from April 2024 until January 2025, in three main governmental hospitals in the West Bank of Palestine (Rafedia hospital in Nablus, Thabet Thabet hospital in Tulkarm and Palestinian Medical Complex in Ramallah). Each of these hospitals has a main pediatric ward.

### Study population, inclusion, and exclusion criteria

The included population is pediatric patients who received antibiotic therapy in the first 6 months of life during hospitalization in the above-mentioned hospitals. After reviewing the medical records, patients who were diagnosed with allergic disease before time of hospitalization, and those which lack the required data regarding the type of antibiotic and parents contact information were excluded. According to UNICEF, the number of new-born children in Palestine in 2023 was 146,578 [15], and the percentage of children diagnosed with allergic disease is 27.2% according to American centers for disease control and prevention [16], so an estimated 39,869 of these children will develop some type of allergic disease. The sample size was estimated at 385, and convenience sampling technique was used in this study.

### Study tools, validity, and reliability

The study had a complete instrument to ensure accurate data collection and analysis. Data collection tools included a questionnaire for the investigation of symptoms related to allergic disease. The data were collected by interview, and prior to any interviews, consent was obtained. Each interview contained identification data: age, gender, and phone number, and the whole questionnaire was covered. The criteria for inclusion and exclusion were, therefore, strict to enhance validity. The questionnaire was reviewed by a pharmacologist and a pediatrician to enhance reliability, and the documentation from the hospitals regarding the antibiotic use of patients in the first six months of life was also reviewed. This questionnaire contained data concerning age, gender, gestational age, class and number of antibiotics used, pets in the house, passive smoking, presence of carpet in the kid’s room, vaccination status, features of the house, the type of infection in the first six months of life, development of and type of allergic diseases. Although infant feeding pattern (breastfeeding, formula, or mixed) was recorded, formula brand and probiotic/prebiotic content could not be reliably recalled by most parents and were therefore excluded from analysis to avoid misclassification.

### Ethics statement

This study was reviewed and approved by the Institutional review board (IRB) at An-Najah National University (Ref: Phram. Med. July. 2024/20). Permission to conduct the study was obtained from the Palestinian ministry of health. Written informed consent attached to the questionnaire and distributed to participants with a detailed description of the study's objectives, as well as its potential benefits and risks. When interview was done using phone calls, participants were also informed about all the previous details. The right to withdraw at any stage was offered. All the information gathered was kept privately and confidentially. The ethical approval was performed in conformity with national and international guidelines to ensure that the study was transparent, with respect for participants' rights and welfare.

### Data analysis

Google Sheets and Microsoft Excel were used for data collection, the data was imported into the program R (version 4.4.2 for windows, by R Core Team) for data analysis. Descriptive and inferential statistics were employed to analyze the data. Frequencies and percentages were reported for categorical variables. The mean and standard deviation were reported for age and weeks of gestation. The chi-square test was used to test the differences between the different groups of antibiotics, A *p*-value of less than 0.05 was considered significant. A complete case analysis was adopted in this study, where we excluded cases with missing data from the final analysis.

## Results

### Sociodemographic and clinical characteristics of the participants

A total of 9000 patient files were viewed, only 1200 children (13.3%) had received antibiotics in the specified time frame, out them only 600 (50%) had correct contact information in order to fill the questionnaire. Of those, parents of only 490 (81.67%) children agreed to fill the questionnaire, 423 (86.33%) filled it correctly, while the other 67 (13.67%) had inaccurate information and were excluded.

The average age of the children was 7.33 ± 1.38 years (mean ± SD), where the ages ranged between 2 and 10. Regarding gestational age, 62 (14.66%) were born preterm (< 37 weeks), 361 (85.34%) were born full term (37–41 weeks) and none were post-term (> 41 weeks). Out of the 423 included children, 264 were males (62.41%). The participants were distributed on 3 cities, Nablus (37%), Ramallah (33%), and Tulkarm (30%).

In terms of feeding patterns, 165 (39.01%) of the children were exclusively breastfed, while 92 (21.75%) were formula-fed, and 166 (39.24%) received both breastmilk and formula. Among the infants who were breastfed, only 23 (5.44%) had allergic symptoms to the milk, whereas allergic symptoms to formula feeding were reported in 22 (5.2%). Regarding environmental exposures, 56 (13.24%) of the participants lived in homes with pets. More than half of the participants were exposed to household smoking (n = 237; 56.03%). The majority of children (n = 394, 93.14%) had a carpet in their rooms. The environmental conditions in the households were also assessed in regard to heating and cooling methods; with electric heating accounted for 36.74% while 16.78% used gas heating and wall-mounted air conditioning units were used in 20.47% of the houses. Central air conditioning was the least common system, found in only 3.36% of households.

We also assessed vaccination status of the patients, with 415 (98.11%) of the children having received all vaccinations to date, while the remaining didn’t receive their vaccinations due to immunodeficiency. Allergic reactions to vaccinations were reported in 20 (4.73%) of cases only. More details are shown in (Table 1).

**Table 1:** Sociodemographic and clinical characteristics of the participants

Age; (mean ± SD)	7.33 ± 1.38 years		
Age group in years, n (%)	≤ 4	9	2.13
	5	41	9.69
	6	33	7.8
	7	168	39.72
	8	65	15.37
	9	100	23.64
	10	7	1.65
Weeks of Gestation; (mean ± SD)	37.5 ± 1.84 weeks		
Weeks of Gestation in weeks, n (%)	Pre-term (< 37 weeks)	62	14.66
	Term (37–41 weeks)	361	85.34
	Post-term (> 41 weeks)	0	0
Gender, n (%)	Male	264	62.41
	Female	159	37.59
Hospital (City)	Nablus	156	36.88
	Tulkarm	129	30.50
	Ramallah	138	32.62
Feeding, n (%)	Breastfeeding	165	39.01
	Formula	92	21.75
	Both	166	39.24
Allergic symptoms while breastfeeding, n (%)	Yes	23	5.44
Allergic symptoms while feeding formula, n (%)	Yes	22	5.2
Pets in the House, n (%)	Yes	56	13.24
Smoker in the House, n (%)	Yes	237	56.03
Carpet in Kid’s room, n (%)	Yes	394	93.14
Vaccination status, n (%)	Yes	415	98.11
Allergic reactions to vaccinations, n (%)	Yes	20	4.73
Heating and conditioning system, n (%) (multiple responses allowed)	Electric heating	219	51.77
	Gas heating	100	26.53
	Wall mounted air conditioning unit	122	32.36
	Fire and wood heating	46	12.20
	Heat and air conditioning unit	28	7.43
	Window air conditioning	27	7.16
	Central air conditioning	20	5.31
	Radiator	1	0.27
	None of the above	33	8.75

### Distribution of allergic symptoms and allergic triggers

Out of the study participants, 125 (29.55%) children have been diagnosed with allergies. In those diagnosed with allergy, the most frequently reported allergy symptoms included redness and itching of the skin (70.4%), wheezing (16.8%), sore throat (10.4%), and shortness of breath (4.8%). In terms of allergens, the most common triggers were food (10.17%), dust (7.33%), insects (7.33%), and cold air (4.96%). Additionally, 4.49% of children had allergic symptoms from triggers/irritants (chemicals/cleaning agents), while a smaller percentage had allergies to trees and grasses (2.6%), wool (0.71%), animals (0.71%), and rubber (0.24%). More details are shown in (Table 2).

**Table 2:** Distribution of allergic symptoms and allergic triggers

	Variable	n	%
Development of allergy	Yes	125	29.55
	No	298	70.45
Common allergy presenting symptoms	Redness and itching of the skin	88	70.4
	Wheezing	21	16.8
	Sore throat	13	10.4
	Shortness of breath	6	4.8
	Local swelling	3	2.4
	Loss of consciousness	2	1.6
	Shortness of breath, general swelling of the body, difficulty swallowing	2	1.6
	Shortness of breath, chills	2	1.6
	Difficulty swallowing	1	0.8
	Chest pain	1	0.8
Type of allergy	No known allergies	290	69.56
	Food	43	10.17
	Insects	31	7.33
	Cold air	21	4.96
	Dust	31	7.33
	Drug	16	3.78
	allergic symptoms from triggers/irritants (chemicals/cleaning agents)*	19	4.49
	Trees and grasses	11	2.6
	Wool	3	0.71
	Animals	3	0.71
	Rubber	1	0.24
	Unknown cause	1	0.24

^*^: for chemicals and cleaning agents, although it was listed in type of allergy, it could be attributed to irritant triggers and not necessarily an IgE-mediated allergy. But these patients had systemic allergy like symptoms, thus it was listed

### Antibiotics use among study participants and the reason for hospitalization

Regarding antibiotic use, the most commonly prescribed antibiotic in the first six months of life was cefotaxime which was used among 332 (78.49%) of the infants followed by ampicillin which was prescribed to 269 (63.59%). Gentamicin (15.13%), amoxicillin (13.24%), and azithromycin (9.46%) were also among the commonly used antibiotics. Less frequently used antibiotics included vancomycin (7.33%), meropenem (4.49%), cefuroxime (3.78%), metronidazole (3.55%), ceftazidime (2.13%), trimethoprim-sulfamethoxazole (1.89%), amikacin (1.65%), cephalexin (1.18%), and erythromycin (0.71%).

When assessing the reason for the hospital admission in our patients, the most common diagnosis was bronchitis (100 cases, 23.64%) followed by pneumonia (60 cases, 14.18%), urinary tract infections (5.44%), meningitis (4.26%), sinusitis (3.07%), and skin infections (2.6%). A small number of cases were diagnosed with ear infections (2.6%), sepsis (2.13%), gastrointestinal infections (1.65%), and yellow fever (0.95%). The largest proportion of cases (167, 39.48%) included infections that were either other types or not clearly identified. More details are found in (Table 3).

**Table 3:** Antibiotics Use Among Study Participants and the Reason for Hospitalization

Antibiotic	n	%
Cefotaxime	332	78.49
Ampicillin	269	63.59
Gentamicin	64	15.13
Amoxicillin	56	13.24
Azithromycin	40	9.46
Vancomycin	31	7.33
Meropenem	19	4.49
Cefuroxime	16	3.78
Metronidazole	15	3.55
Ceftazidime	9	2.13
Trimethoprim-Sulfamethoxazole	8	1.89
Amikacin	7	1.65
Cephalexin	5	1.18
Erythromycin	3	0.71
*Reason for Hospitalization*		
Bronchitis	100	23.64
Pneumonia	60	14.18
Urinary tract infection	23	5.44
Meningitis	18	4.26
Sinusitis	13	3.07
Skin infections	11	2.6
Gastrointestinal infections	7	1.65
Ear infections	11	2.6
Sepsis	9	2.13
Yellow fever	4	0.95
Joint infection	4	0.95
Immunodeficiency	2	0.47
Burn	1	0.24
Other/not identified	167	39.48

### Relationship between antibiotics and allergies

A Chi-square test of independence was performed to examine the relationship between the number of antibiotic classes used in the first 6 months of life and developing allergies, the Chi-square statistic was 2.64, p-value 0.450, degree of freedom 3. With the formula *X*^2^ (3, *N* = 423) = 2.64, *p* = 0.450. Since the p-value is greater than 0.05, we failed to reject the null hypothesis, therefore, there was no statistically significant association between the number of antibiotic classes used in the first 6 months of life and developing allergies in our dataset. (Table 4).

**Table 4:** The relationship between number of antibiotic types used and developing allergies

Number of Antibiotic classes	Didn’t develop allergies	Developed allergies	Total
1 Antibiotic	59 (69.4%)	26 (30.6%)	85 (100%)
2 Antibiotics	177 (68.6%)	81 (31.4%)	258 (100%)
3 Antibiotics	39 (79.6%)	10 (20.4%)	49 (100%)
4 + Antibiotics	23 (74.2%)	8 (25.8%)	31 (100%)

We used Firth logistic regression to analyze the relationship between the exposure to different types of antibiotics in the first six months of life, and developing allergies, the dependent variable was allergy status (yes–no), and the primary independent variable was antibiotic class, we included age as a continuous covariate to control the potential confounding effect.

**Fig. 1:**
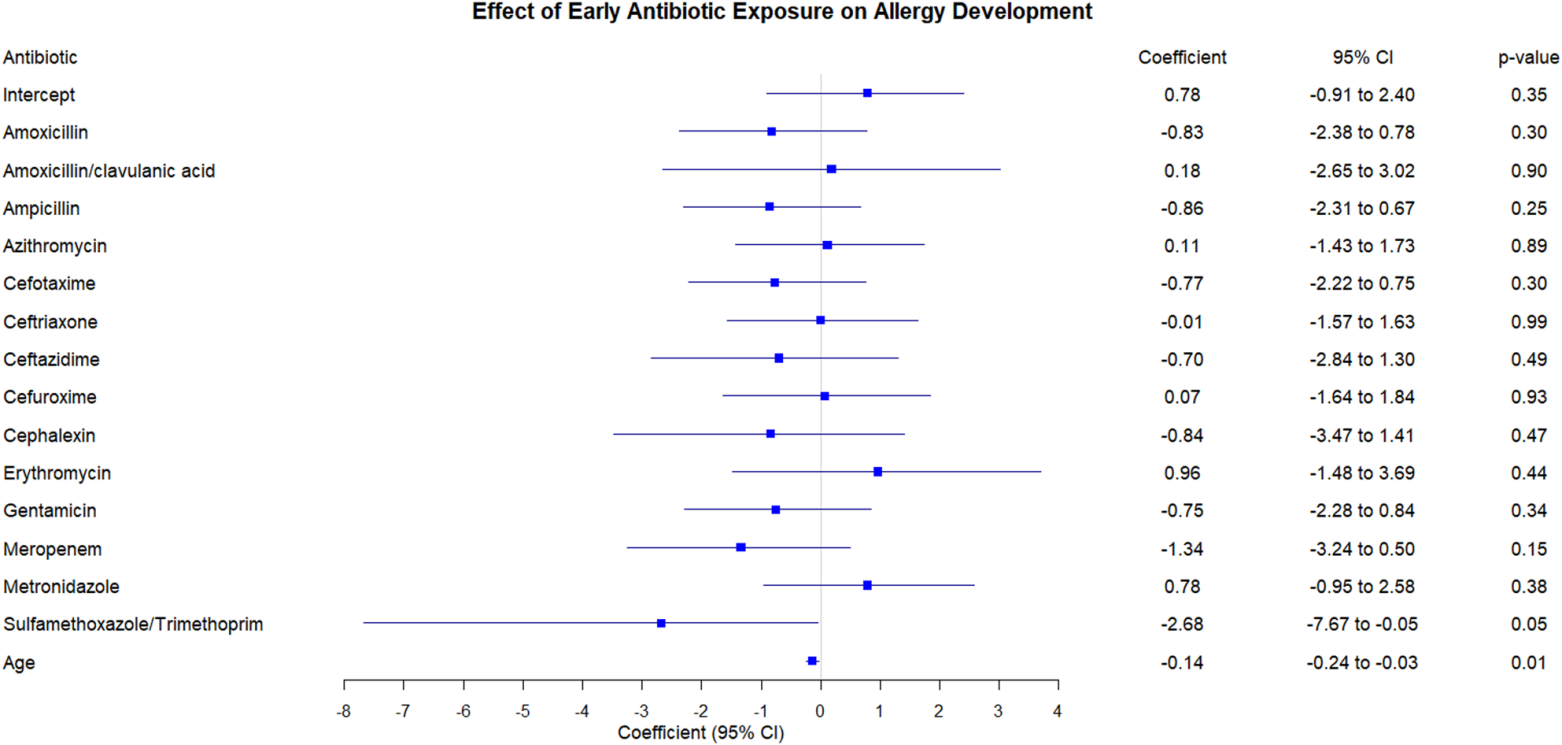
Forest plot of the different types of antibiotics used in the first 6 months of life and developing allergies

Most antibiotics did not exhibit a statistically significant association with allergy (*p* > 0.05). TMP-SMX was associated with lower odds of allergy; however, estimates are imprecise due to small exposure counts and should be considered hypothesis-generating. Age was also a significant predictor, with lower odds of allergy with increasing age (Table 5, Fig. 1).

**Table 5:** The relationship between exposure to different types of antibiotics in the six months of life and developing allergies

Variable	Coefficient	SE (Coefficient)	Lower 0.95	Upper 0.95	Chisq	*p*-value
(Intercept)	0.78	0.82	− 0.91	2.4	0.86	0.35
Amoxicillin	− 0.83	0.78	− 2.38	0.78	1.09	0.3
Amoxicillin/clavulanic acid	0.18	1.36	− 2.65	3.02	0.02	0.9
Ampicillin	− 0.86	0.73	− 2.31	0.67	1.31	0.25
Azithromycin	0.11	0.78	− 1.43	1.73	0.02	0.89
Cefotaxime	− 0.77	0.73	− 2.22	0.75	1.07	0.3
Ceftriaxone	− 0.01	0.79	− 1.57	1.63	0	0.99
Ceftazidime	− 0.7	1.03	− 2.84	1.3	0.48	0.49
Cefuroxime	0.07	0.87	− 1.64	1.84	0.01	0.93
Cephalexin	− 0.84	1.18	− 3.47	1.41	0.52	0.47
Erythromycin	0.96	1.26	− 1.48	3.69	0.6	0.44
Gentamicin	− 0.75	0.77	− 2.28	0.84	0.93	0.34
Meropenem	− 1.34	0.93	− 3.24	0.5	2.08	0.15
Metronidazole	0.78	0.88	− 0.95	2.58	0.78	0.38
Sulfamethoxazole/Trimethoprim	− 2.68	1.62	− 7.67	− 0.05	4.01	**0.05**
Age	− 0.14	0.05	− 0.24	− 0.03	6.46	**0.01**

## Discussion

In this multi-center retrospective cohort of 423 children who received antibiotics within the first six months of life, 29.6% were reported to have allergic disease at follow-up. Given the retrospective design and sampling frame restricted to antibiotic-exposed hospitalized infants, these findings should be interpreted as associations rather than evidence of causation. Unlike population-based birth cohorts that include unexposed comparators, our study did not include a “no-antibiotic” group, which limits causal inference and may partly explain differences from some prior cohorts. Neither the number of antibiotics received nor the class of antibiotic was significantly associated with developing allergy, except for TMP-SMX, which showed lower odds of developing allergy. We also noticed a decline of allergy prevalence with increasing age. Because our cohort includes only antibiotic-exposed hospitalized infants and lacks an unexposed comparator, our analysis evaluates class-level differences within an exposed cohort rather than the overall effect of antibiotic exposure versus no exposure. Nevertheless, population-based cohorts have reported higher risks of asthma or atopic dermatitis following early-life antibiotic exposure, particularly with broad-spectrum agents in the first year of life [17]. A 2023 systematic review (58 studies, > 6 million children) calculated a pooled odds ratio of 1.22 (95% CI, 1.15–1.30) for any allergy after ≥ 1 antibiotic course in infancy, but highlighted substantial heterogeneity by drug class and study design [18].

Our finding of no association between early life use of β-lactams and macrolides in developing allergy compared to other classes echoes the findings of a similar study done in Finland [17]. Moreover, our study is the first in the Middle-East to look at how different classes of antibiotics may relate to allergy risk.

The finding of decreased allergy with use of TMP-SMX can be attributed to several factors, first is that it was found to be microbiome sparing, supported by in-vitro and in-vivo data, as it produced minimal, short-lived shifts in α‑diversity in comparison with macrolides and cephalosporins, since the latter two cause prolonged depletion of Bifidobacterium and Lactobacillus spp. [19]. Another study in regards to pediatric prophylaxis found that gut microbiome diversity remained stable after 6 months of low-dose TMP-SMX, while also having a selective suppression of Enterobacterales, such as Escherichia coli and Klebsiella species, which are the main causative bacteria of febrile urinary tract infections, but with preservation of commensal Clostridiales, which are key inducer of regulatory T (Treg) cells [20]. Second is the direct immunomodulation effect of TMP-SMX, as recent work demonstrated that it can augment mast-cell-dependent IL‑10 and IFN‑γ production, promoting a T_H1‑skewed milieu and tumor cytotoxicity, such a shift away from T_H2 bias could theoretically dampen IgE‑mediated allergy [21]. 

Regarding the decreased allergy prevalence with increasing age, other longitudinal natural-history studies showed that IgE sensitization peaks in early childhood, and it partially remits by late adolescence as in one study regarding food allergy, 70% of the milk and egg allergies had a remission [22].

This occurs possibly due to the maturation of the gut and airway regulatory networks, which expands Treg pools and promotes class switch form IgE to IgG4, and the diversification of the microbiology’s as the child’s diet broadens, and also the allergen-specific immunotherapy that occurs due to natural exposure, especially for aeroallergens, thus leading to desensitization. Our finding of a 14% relative reduction in allergy odds per additional year corroborates these established trajectories.

## Conclusion

The prevalence of allergy among children exposed to antibiotics in the first six months of life was approximately one third. We found no significant association between the number of antibiotic classes and later allergy. TMP-SMX showed a hypothesis-generating signal toward lower odds of allergy, but estimates were imprecise due to small exposure counts. Further prospective studies including an unexposed comparator group, larger sample sizes, and detailed infection history are needed to clarify these associations.

## The strengths and limitations of the study

This study offers a number of advantages, including a focus on Palestinian pediatric patients, which can offer valuable insights into the relationship between early antibiotic exposure and the appearance of allergies. The clinical significance of the findings may be extensively examined by reviewing the medical records using retrospective data analysis. The limits of this study are that antibiotic exposure might be affected by recall and reporting bias. Genetic and environmental confounding was not fully accounted for. There may be limited generalizability due to healthcare and prescribing practices. Given the retrospective design, there is also limited ability to establish a causal relationship between antibiotic use and allergies. Detailed information on formula brand and probiotic/prebiotic composition was unavailable and may contribute to residual confounding. Finally, our study lacks a control group, thus limiting causal inference and preventing direct estimation of excess risk attributable to antibiotics versus no exposure, but it builds the grounds for further prospective studies on this very important topic affecting all children.

We also faced many difficulties in obtaining the data for this study, starting with poor documentation of the doses of the given medications, as the health system only involves medications given by the ministry of health hospitals and centers, where a national health system that collects the complete history of the patient should be implemented, including medications taken from external pharmacies, as such change will improve the quality for the patient and caregiver.

## Recommendations

Due to these limitations, further research is required through prospective cohorts of larger and more diverse populations to understand the issues of causality better. In addition, health professionals should be encouraged to adhere to guidelines in antibiotic stewardship in order to avoid unnecessary prescriptions, especially in young children. Public awareness through campaigns will increase awareness in parents about the risks of overuse and alternative treatments when necessary. Further research into the effects of antibiotics on gut microbiota can perhaps give a new direction to the strategy for the prevention of allergy. And lastly, the development of national policies that address antibiotic prescribing in pediatric practices, as well as prudent use within health institutions, needs serious attention from the concerned policymakers.

## Data Availability

The datasets used and/or analyzed during the current study available from the corresponding author on reasonable request.
